# Identification of Cell-Binding Adhesins of *Leptospira interrogans*

**DOI:** 10.1371/journal.pntd.0003215

**Published:** 2014-10-02

**Authors:** Karen V. Evangelista, Beth Hahn, Elsio A. Wunder, Albert I. Ko, David A. Haake, Jenifer Coburn

**Affiliations:** 1 Graduate Program in Microbiology, Immunology, and Molecular Genetics, Medical College of Wisconsin, Milwaukee, Wisconsin, United States of America; 2 Division of Infectious Diseases, Department of Medicine, Medical College of Wisconsin, Milwaukee, Wisconsin, United States of America; 3 Department of Epidemiology of Microbial Diseases, Yale School of Public Health, New Haven, Connecticut, United States of America; 4 Division of Infectious Diseases, VA Greater Los Angeles Healthcare System, Los Angeles, California, United States of America; 5 Departments of Medicine, Urology, and Microbiology, Immunology, and Molecular Genetics, David Geffen School of Medicine at UCLA, Los Angeles, California, United States of America; University of Tennessee, United States of America

## Abstract

Leptospirosis is a globally distributed bacterial infectious disease caused by pathogenic members of the genus *Leptospira*. Infection can lead to illness ranging from mild and non-specific to severe, with jaundice, kidney and liver dysfunction, and widespread endothelial damage. The adhesion of pathogenic *Leptospira* species (spp.), the causative agent of leptospirosis, to host tissue components is necessary for infection and pathogenesis. While it is well-established that extracellular matrix (ECM) components play a role in the interaction of the pathogen with host molecules, we have shown that pathogenic *Leptospira interrogans* binds to host cells more efficiently than to ECM components. Using *in vitro* phage display to select for phage clones that bind to EA.hy926 endothelial cells, we identified the putative lipoproteins LIC10508 and LIC13411, and the conserved hypothetical proteins LIC12341 and LIC11574, as candidate *L. interrogans* sv. Copenhageni st. Fiocruz L1–130 adhesins. Recombinant LIC11574, but not its *L. biflexa* homologue LBF1629, exhibited dose-dependent binding to both endothelial and epithelial cells. In addition, LIC11574 and LIC13411 bind to VE-cadherin, an endothelial cell receptor for *L. interrogans*. Extraction of bacteria with the non-ionic detergent Triton X-114 resulted in partitioning of the candidate adhesins to the detergent fraction, a likely indication that these proteins are outer membrane localized. All candidate adhesins were recognized by sera obtained from leptospirosis patients but not by sera from healthy individuals as assessed by western blot. This work has identified bacterial adhesins that are potentially involved in *L. interrogans* infection of the mammalian host, and through cadherin binding, may contribute to dissemination and vascular damage. Our findings may be of value in leptospirosis control and prevention, with the bacterial adhesins potentially serving as targets for development of diagnostics, therapeutics, and vaccines.

## Author Summary

Leptospirosis, caused by pathogenic species of the genus *Leptospira*, is an infectious disease that has emerged as a globally important health problem. Infection can either lead to mild illness or can progress to a severe disease form manifested by jaundice, kidney and liver dysfunction, and widespread blood vessel damage. It is thought that the ability of the bacteria to recognize and bind to human and animal cells is important for *Leptospira* spp. to cause the disease. Using phage display, we were able to identify bacterial proteins that mediate the binding of the bacteria to host cells. One of the identified proteins, LIC11574, attaches to different types of host cells, and to VE-cadherin, a cell surface protein previously identified as receptor for disease-causing *L. interrogans*. All bacterial proteins identified were recognized by sera obtained from leptospirosis patients but not by sera from healthy individuals. Our findings may be of value in leptospirosis control and prevention, with these bacterial surface proteins as new targets for serodiagnosis and vaccine development.

## Introduction

Leptospirosis, caused by pathogenic species of the genus *Leptospira*, is the most widespread zoonosis and has emerged as a major public health problem worldwide, particularly in the humid tropical and subtropical areas in developing countries. The clinical manifestations of human leptospirosis are diverse, ranging from mild illness to a severe disease form known as Weil's syndrome, characterized by jaundice, acute renal and hepatic failure, pulmonary distress, and hemorrhage. Specific groups at risk for infection include farmers, sewer workers, slaughterhouse workers, and veterinarians [1]. Additionally, outbreaks of this disease are associated with severe flooding events particularly in urban slum areas [2]–[4]. During infection, *Leptospira* spp. can rapidly disseminate from the portal of entry to any tissue of the host, and persist in the proximal tubules of the kidney of reservoir hosts such as rodents. Leptospirosis is spread through the contact of mucous membranes, cuts, or abrasions with fresh water contaminated by the urine of infected animals [5]–[7].

The mechanisms involved in leptospiral pathogenesis are not well elucidated. However, it is generally thought that the adhesion of *Leptospira* spp. to host tissue components is a necessary step for infection and pathogenesis. Attachment to host cells, and extracellular matrix (ECM) components, is likely to be necessary for the ability of leptospires to penetrate, disseminate and persist in mammalian host tissues. It has been demonstrated that pathogenic *Leptospira* binds to a variety of cell lines including fibroblasts, epithelial, endothelial, microglial and monocyte/macrophage [8]–[16], and ECM components [17]–[25] *in vitro*.

Many pathogenic bacteria express adhesive molecules (adhesins) on their surfaces that promote interaction with host cell receptors or with other macromolecules. Adhesins are surface-exposed proteins, examples of which include intimin in *Escherichia coli* [26] and invasin in *Yersinia pseudotuberculosis* [27]. A number of *L. interrogans* proteins have been shown to bind different ECM components *in vitro*, including leptospiral immunoglobulin (Ig)-like repeat proteins LigA and LigB [20], [22], [24], [28]–[31], LenA [21], and OmpL1 [25]. OmpL1, a 31-kDa transmembrane outer membrane protein (OMP), binds to laminin, plasma fibronectin, and plasminogen [25], [32], [33].

Although it is well established that ECM components play a role in the interaction of the pathogen with host molecules [17], [20], [21], [23]–[25], [30], [34], recent data generated in our laboratory show that pathogenic *Leptospira* bind host cells more efficiently *in vitro* [8], [35]. Attachment of *L. interrogans* to endothelial cell monolayers is mediated at least in part by the host cell surface protein VE-cadherin [35], although the leptospiral adhesin(s) involved in this interaction remained unknown.

In this study, we identified *L. interrogans* proteins involved in interactions with host cells using phage display. Phage display has been successful in identifying adhesins such as P66 in another spirochete, *Borrelia burgdorferi* [36], [37]. This work also describes the characterization of the binding properties of the candidate adhesins, and provides evidence that these proteins are expressed during *Leptospira* infection.

## Materials and Methods

### Ethics statement

All work with animals was performed under guidelines of the Public Health Services Policy on Humane Care and Use of Laboratory Animals.

Mice were euthanized by controlled CO2 administration followed by cardiac puncture.

The Institutional Animal Care and Use Committee of the Medical College of Wisconsin approved all work with animals. The protocol number is AUA2391.

### Animals

BALB/c mice were purchased at the age of 3 weeks from Charles River Laboratories (Wilmington, MA). Mice were fed and watered *ad libitum* throughout the course of the experiment.

### Bacterial strains

*Leptospira interrogans* serovar Copenhageni (pathogenic, strain Fiocruz L1–130) was reisolated by infection of hamsters, and then stored at passage 1 and 2 in liquid nitrogen. Frozen aliquots were thawed and passaged in liquid Ellinghausen-McCullough-Johnson-Harris (EMJH) medium [6] supplemented with 5-fluorouracil (Sigma-Aldrich, St. Louis, MO) and 10% heat-inactivated rabbit serum (Gibco, Grand Island, NY) at 30°C. This strain has a 50% lethal dose range of 37–10^4^ in hamsters [38]–[40] and the genome sequence was previously reported [41]. *L. biflexa* serovar Patoc (avirulent; strain 23582) was obtained from the American Type Culture Collection (ATCC) (Manassas, VA) and grown in EMJH medium. Genomic DNA used to construct the phage library was obtained from growing 1 liter of *L. interrogans*. *L. interrogans* DNA was phenol- and chloroform-extracted, ethanol-precipitated and resuspended in 100 µl Tris-EDTA pH 8.0 as previously described [42].

*Escherichia coli* strain MC1061 (F^−^) was used as the host for the generation of the phage display library while *E. coli* strain TG1 (F^+^) was used to recover selected phage clones through infection. *E. coli* strain Top10 was utilized for gene cloning, and *E. coli* strain KS330 was used for protein expression and purification. *E. coli* were grown in Luria-Bertani (LB) or 2x YT medium (BD, Sparks, MD) supplemented with 0.2% v/v dextrose at 30°C with shaking. For plating, the media were supplemented with 15 g/l agar. Antibiotics (tetracycline, 12.5 µg/ml, ampicillin, 100 µg/ml and chloramphenicol, 30 µg/ml) (Sigma-Aldrich) were added as appropriate.

### Mammalian cell lines

Human epithelial and endothelial cell lines were used in this study, as these represent cell types that are relevant to *Leptospira* spp. infection. The human laryngeal epithelial cell line HEp-2 was purchased from the ATCC, and grown in Eagle's Minimum Essential Medium (ATCC) supplemented with 10% fetal bovine serum (FBS) (Gibco, Grand Island, NY) at 37°C under 5% CO_2_. The human macrovascular endothelial cell line EA.hy926, a kind gift from Dr. C. J. Edgell (University of North Carolina, Chapel Hill, NC) [43], [44], was grown at 37°C under 5% CO_2_ in Dulbecco's modified Eagle medium (DMEM) (Gibco) with 4.5 g/l glucose, supplemented with 10% FBS, 100 µM hypoxanthine-0.4 µM aminopterin-16 µM thymidine (Sigma-Aldrich), and 2 mM L-glutamine (Gibco). The human microvascular endothelial cell line HMEC-1 [45] obtained from the Centers for Disease Control and Prevention (CDC), was grown in MCDB 131 medium (Gibco) and supplemented with 15% heat-inactivated FBS (Hyclone, Logan, UT), 2 mM L-glutamine (Gibco), 10 ng/ml epidermal growth factor (EGF) (BD Biosciences, Bedford, MA), 1 µg/ml hydrocortisone (Sigma) and 25 mM HEPES (Gibco). All cell lines were grown in the presence of 100 U/ml penicillin and 100 µg/ml streptomycin (Gibco).

### Construction of the *L. interrogans* phage display library

The display system used in this study is based on in-frame fusions to the phage minor coat protein encoded by gene III. The vector used to construct the filamentous phage library is derived from fdDOG [46] inserted with a stuffer region containing stop codons in all three reading frames upstream of gene III and flanked by *Sfi*I restriction sites [37]. The resultant vector, fdBUG, cannot express the minor coat protein which is necessary for infecting naïve F^+^ *E. coli* cells, causing a reduction in infectious phage particle production [37]. The phage vector fdBUG was digested with *Sfi*I (New England Biolabs, Ipswich, MA) for 4 hr at 50°C, which removes the stuffer region and leaves 3′-GTG overhangs. To prevent self-ligation, the digested vector was treated with alkaline phosphatase (calf intestinal, New England Biolabs) for 1 hr at 37°C.

*L. interrogans* genomic DNA was partially digested for 1 hr at 37°C using *Mse*I (New England Biolabs), which leaves a 5′-TA overhang. The genomic DNA fragments were ligated with pre-annealed synthetic oligonucleotides (Table 1) at a molar ratio of 2 equimolar mixture of adaptors to 1 genomic DNA using T4 ligase (New England Biolabs). The three adaptors each have 5′-TA overhangs cohesive with the partially digested *L. interrogans* DNA at one end, and 3′-CAC overhangs cohesive with the ends left by *Sfi*I digestion of the vector at the other end as previously described [37]. The adapted DNA was fractionated by agarose gel electrophoresis and fragments between 0.2 and 1 kilo base pairs (kbp) were extracted using a commercially available gel extraction kit (QIAquick Gel Extraction kit, QIAGEN, Germantown, MD). The size-selected *L. interrogans* DNA fragments were ligated with *Sfi*I-digested, dephosphorylated fdBUG vector (20 insert: 1 vector molar ratio) using T4 ligase. The resulting ligation mixture was electroporated into *E. coli* MC1061 cells and selected on LB agar supplemented with 12.5 µg/ml tetracycline. As a control, fdBUG was transformed into *E. coli* MC1061. The presence of *L. interrogans* DNA inserts in transformants were assessed by performing colony PCR as previously described [37] on twenty-four randomly picked colonies per transformation using primers oDOG1 and oDOG5 (Table 1), which amplify the region flanked by the two *Sfi*I restriction sites.

**Table 1:** Oligonucleotides used in this study.

Oligonucleotides (5′→3′)		Source/Reference
***Adaptors used to construct the phage display library of L. interrogans***		Coburn *et al.*, 1999 [37]
oJLC13	GCTGGTTGGCCAAAG	
oJLC14	TACTTTGGCCAACCAGCCAC	
oJLC15	GCTGGTTGGCCAAAAG	
oJLC16	TACTTTTGGCCAACCAGCCAC	
oJLC17	GCTTGGTTGGCCAAAAG	
oJLC18	TACTTTTGGCCAACCAAGCCAC	
***Phage vector sequences used for PCR amplification of inserted DNA***		Coburn *et al.*, 1999 [37]
oDOG1	GTCGTCTTTCCAGACGTTAGT	
oDOG5	CCTTTCTATTCTCACAGTGCACAGGTCC	
***Vector pMalC2 sequences for PCR amplification of insert DNA***		Coburn *et al.,* 1999 [37]
oMal01	CGCTTTCTGGTATGCCGTGCGTA	
oMal02	TCTCATCCGCCAAAACAGCCAAG	
***Cloning and sequencing of candidate adhesins to generate MBP fusions***		This study
LIC10508 For	GGTTCTAGAAAGAGCAAAGAAGAAATTGAAAGAG	
LIC10508 Rev	TTGTCGACCTAACAACCAGGACCTTCACATGC	
LIC10508 oF3	GATCGAGAAGCCAGTATGAAATTAG	
LIC10508 oR4	GCTTCAACACGAACTTCATAACG	
LIC11574 For	AGTTCTAGAGCAAACTTTATCATTCCTTCG	
LIC11574 Rev	CAGTCGACTTAAGGCTGTTGTTGTTCG	
LIC11574 oF3	ACCTATCTTGGGATCTCGGCAG	
LIC11574 oR4	CTTCTTTAATTCCACTTTCGTCTCG	
LIC12341 For	CTTATCTAGACAAACTTCGACGGAAAATTCTAC	
LIC12341Rev	ATGTCGACTTAATTTTTTGCTTCTTTTTTAATAAGAGC	
LIC12341 oR5	TCCTTGCGGTCAGATTCTCC	
LIC12341 oF6	CAATCGCTCATAAATACTTCG	
LIC13411For	TGTGGATCCTATTTTCAAAATAGAAAAAACG	
LIC13411Rev	GGTCTGCAGTTTCTAATCCGCAACGTC	
LIC13411 oR3	AACCTCCTCGAAGTCCATAACC	
LIC13411 oF4	GAAACAACTTCTTCGGAAACTACG	
OmpL1For	TTCAGGATCCAAAACATATGCAATTGTAGGATTTGG	
OmpL1Rev	TTGTCGACCTAACAACCAGGACCTTCACATGC	
LBF1629For	AGGTGGATCCCGAAACCTCATCATCCCTTCCACC	
LBF1629Rev	CCTTCTGCAGTTAACGAGGTTGTGTTTTTGCATTGG	

Underlined are the sequences recognized by restriction enzymes *Xba*I (TCTAGA), *Sal*I (GTCGAC), *Bam*H1 (GGATCC), and *Pst*I (CTGCAG).

### Selection of phage clones that bind to cells

Three rounds of selection for phage that bind to EA.hy926 cells were performed using five independent pools of the phage library. Phage particles from 100 ml overnight cultures were prepared as described previously [36], [37] with the exception that the phage were resuspended in DMEM supplemented with 1% BSA and protease inhibitors 1 mM benzamidine and 0.01 trypsin inhibitory units (TIU)/ml aprotinin (2PI) (Sigma-Aldrich). To eliminate clones that bind to ECM, the phage library was initially pre-adsorbed on ECM. ECM was obtained from EA.hy926 cells grown in a 6-well plate (BD Falcon, Bedford, MA). Once the monolayers reached confluence, medium was removed and cells were washed with PBS. Two milliliters of 10 mM EDTA were added to each well and incubated for 10 min at RT on a rocker to lift the cells. The cells were removed by aspiration and the plates were visually assessed under a microscope to ensure complete removal of cells. The ECM left on the plate was washed three times with HBSC buffer (25 mM HEPES, pH 7.8, 150 mM NaCl, 1 mM MnCl_2_, 1(mM MgCl2, 250((M CaCl2) prior to pre-adsorption of phage.

Six ml of a 1(100 dilution of the phage suspension (approximately 1013 phage particles) were added to ECM and incubated for 3 hr at 37°C under 5% CO2. The unbound phage suspension was collected and added to 6-well plates containing confluent EA.hy926 monolayers pre-washed three times with HBSC. After 2 hr of incubation at 37°C under 5% CO_2_, the unbound phage particles were removed and the wells were washed 15x with HBSC. The phage clones that specifically bound to intact monolayers were eluted using 3 ml of glycine pH 2.2, and pH was then adjusted to pH 7.4 with 1 M Tris pH 10. The eluted phage particles were used to infect *E. coli* strain TG1. The infected clones were selected by overnight growth at 30°C on LB plates supplemented with 12.5 µg/ml tetracycline. All colonies obtained from each elution step were pooled and used to make new phage stocks for the second round of selection, performed as described. The same steps were repeated for the third round of selection, after which the selected pools were kept at −80°C until further use. As a control, phage particles were also prepared from vector fdDOG, which has an intact gene III, and therefore can produce the minor coat protein and so is capable of infecting bacterial cells. Three rounds of selection for fdDOG were performed in parallel with the phage library.

### Analysis of selected phage clones

After 3 rounds of selection, individual colonies of phage clones in *E. coli* TG1 were purified by plating pooled phage clones, then restreaking on LB plates supplemented with tetracycline. Individual colonies were picked and colony PCR was performed using primers oDOG1 and oDOG5 (Table 1). The amplified products were sent for sequencing (McLab, South San Francisco, CA). The sequences obtained were compared by Basic Local Alignment Search Tool (BLAST) with the complete genome of *L. interrogans* sv. Copenhageni st. Fiocruz F1-130 at the NCBI site (http://blast.ncbi.nlm.nih.gov) to identify open reading frames to which the selected DNA fragment corresponded. The *L. interrogans* DNA inserted in the phage genome was also checked for correct gene orientation and intact reading frame. The genes identified were analyzed for the presence of signal peptide using online versions of prediction programs SignalP 3.0 (http://www.cbs.dtu.dk/services/SignalP-3.0) and LipoP 1.0 (http://www.cbs.dtu.dk/services/LipoP). The β-barrel prediction program PRED-TMBB (http://biophysics.biol.uoa.gr/PRED-TMBB) was utilized to determine if any of the gene hits might encode transmembrane OMPs. The predicted protein sequences were also manually analyzed for the presence signal peptides recognized by the spirochaetal lipoprotein signal peptidase (Lsp) or leader peptidase (Lep) [47]. Selected phage clones containing fragments of genes that are predicted to encode proteins that contain secretion signal sequences were further assessed based on the following criteria: (a) their known or putative surface localization (predicted or shown experimentally), (b) the presence of a predicted transmembrane β-barrel motif, and (c) whether they have homologues in pathogenic and/or saprophytic strains.

### Cloning, expression and purification of recombinant *Leptospira* candidate adhesins

The genes encoding the *Leptospira* candidate adhesins LIC10508, LIC11574, LIC12341, and LIC13411 identified by phage display, LBF1629, which encodes the LIC11574 homologue in *L. biflexa*, and a known *L. interrogans* ECM adhesin OmpL1, were cloned in the expression vector pMalC2 (New England Biolabs) and expressed as fusions to maltose binding protein (MBP). The *Leptospira* genes without the sequences encoding the signal peptides were amplified by PCR using *L. interrogans* sv. Copenhageni st. Fiocruz F1–130 genomic DNA as template. The oligonucleotide primers containing restriction sites used in this study are listed in (Table 1). The amplified products were cleaved with appropriate restriction enzymes to generate non-compatible cohesive overhangs to facilitate directional cloning when ligated to pMalC2 digested with the same enzymes. The ligation products were transformed into *E. coli* strain Top10 by electroporation. The transformants were selected on LB plates supplemented with 100 µg/ml ampicillin. Plasmids from selected colonies were isolated using a commercially available kit (Plasmid Mini Kit, QIAGEN) and the presence of insert DNA was verified by PCR using vector specific primers oMal01 and oMal02 (Table 1), which hybridize to vector DNA sequences flanking the insertion site. Plasmid DNA was also sequenced in a commercial facility (McLab, San Francisco, CA) using oMal01, oMal02 and custom-designed internal gene primers (Table 1). The plasmids with appropriate inserts were transformed into *E. coli* expression strain KS330.

The production of MBP-fusions from 1 liter *E. coli* cultures in 2x YT was induced with 1 mM isopropyl-β-D-thiogalactopyranoside (IPTG) (Sigma-Aldrich) for 2 hr at 30°C as previously described [48], and assessed by silver staining and by immunoblot analysis using an α-MBP antibody. Bacterial cells were harvested by centrifugation and lysates were prepared by pressing the cells at 10,000 psi. The recombinant proteins were purified from the supernatant by amylose affinity chromatography. The protein concentrations of MBP fusions were determined using a Bradford method-based protein assay kit (Bio-Rad, Hercules, CA).

### *In vitro* binding assays

To test the binding of purified phage particles or recombinant MBP fusions to cell monolayers, the epithelial cell line HEp-2, and endothelial cell lines EA.hy926 or HMEC-1, were seeded in 96-well tissue culture plates (BD Falcon) at 50% confluence and incubated at 37°C, 5% CO_2_ for 2 days until cells were confluent. In some cases, the cells were incubated for an additional 1–2 days to reach post-confluence, while cell viability was maintained. Controls for binding included incubation of phage particles or recombinant proteins in medium alone (binding to plastic) or in ECM prepared from the cell lines as described above.

To test the ability of the phage particles to bind to cell monolayers, purified phage particles were prepared from individual selected clones as previously described [37]. Approximately 10^10^ phage particles resuspended in 50 µl DMEM supplemented with 1% BSA and protease inhibitors benzamidine and aprotinin were added to confluent monolayers in quadruplicate on a 96-well tissue culture plate. The plates were incubated for 2 hr at 37°C under 5% CO_2_ to allow phage attachment. The unbound phages were removed by washing with HBSC, while the bound phages were fixed with 3% PFA in PBS. After washing with Tris-buffered saline (TBS), the phage particles bound to monolayers or ECM were detected using an enzyme linked immunosorbent assay (ELISA)-based assay. Briefly, wells were blocked for 1 hr with 1% BSA in HBSC. The plates were washed 3x with TBS, then incubated with rabbit anti-M13+ fd bacteriophage antibody (diluted 1∶10,000; Abcam, Cambridge, MA). After 1 hr, the plates were washed 3x with TBS, followed by incubation with anti-rabbit IgG horseradish peroxidase (HRP)-conjugated antibody (diluted 1∶10,000; Promega) for 40 min at RT. The blocking step and incubations with antibodies were performed at RT on a rocker. Plates were developed for 30 min with 3,3′,5,5′-Tetramethylbenzidine (TMB, Sigma-Aldrich) and 0.006% hydrogen peroxide in phosphate-citrate buffer (0.05 M Na_2_HPO_4_, 0.02 M citric acid, pH 5.0) for 30 min. The absorbance at 655 nm was determined using an automated plate reader (SpectraMax M5, Molecular Devices). Results were expressed as fold difference in the absorbance readings in the presence of cells over no cells.

To test the binding of recombinant *Leptospira* proteins to cells *in vitro*, cell monolayers were incubated with various concentrations (ranging from 0.1–3.0 µM) of MBP fusion proteins in DMEM supplemented with 1% BSA, 25 mM HEPES and 2PI for 1 hr at 37°C under 5% CO_2_. The addition of recombinant proteins was done in quadruplicate wells. The plates were washed with 200 µl HBSC to remove the unbound proteins and fixed with 3% PFA in PBS. The wells were washed once with TBS, then the recombinant proteins bound to host cells were measured using an ELISA format. The wells were blocked with 1% BSA in TBS with 3% goat serum, washed, and then incubated with rabbit anti-MBP antibody (diluted 1∶10,000; New England Biolabs, Beverly, MA) for 1 hr at RT. After washing, HRP-conjugated anti-rabbit antibody (diluted 1∶10,000; Promega) was added to each well and incubated for 40 min at RT under constant shaking. Wells were developed for 25 min with TMB and 0.006% hydrogen peroxide in 0.05 M phosphate-citrate buffer (pH 5.0) and absorbance at 655 nm was read as described previously. The cell-specific attachment was determined by subtracting the absorbance signal of recombinant proteins in empty wells/plastic from the absorbance in wells containing cells, with each assay done in quadruplicate.

The attachment of recombinant proteins to purified receptors was also tested using the ELISA described above. The wells of pre-chilled 96-well Linbro plates (MP Biomedicals, OH) were coated with 50 µl/well 0.01 µM purified VE-cadherin (Human CD-144, cadherin-5) obtained from Bioclone Inc. (San Diego, CA) and diluted in HBSC supplemented with 2PI in quadruplicate wells. The amount of receptor used to coat wells was optimized and described previously by our group [35]. The plates were incubated overnight at 4°C with shaking. The controls used in the assay included HBSC buffer alone or BSA, coated at the same concentration as VE-cadherin. The plates were washed with HBSC and blocked with 1% BSA in DMEM for 1 h at 4°C, prior to the addition of 0.001–1 µM recombinant adhesins. The addition and detection of MBP fusion proteins on plates were carried out as described above. Receptor binding was expressed as the difference in the absorbance (OD_655_) readings between empty and VE-cadherin-coated wells.

### Recognition of recombinant proteins by sera from leptospirosis patients

Sera used for this evaluation were from de-identified banked samples obtained from subjects enrolled in protocols approved by the Institutional Review Board (IRB) committees of Yale University and Oswaldo Cruz Foundation. Convalescent-phase sera were obtained from Brazilian patients with confirmed leptospirosis [49]. Sera from control subjects were obtained from individuals residing in urban slum communities from the same site [4]. The serum from each individual at 1∶500 dilution was pre-cleared by incubation with a PVDF membrane blotted with whole cell lysate of *E. coli* expressing MBP-β-galactosidase. The purified recombinant proteins were separated by 10% SDS-PAGE and transferred to PVDF membranes for western blotting. The membranes were blocked with TBS containing 5% nonfat dry milk overnight at 4°C. After 3 washes with TBS, the membranes were incubated in pre-cleared human serum for 3 hr at RT on a rocker. The membranes were washed 3x with 1x TBS before probing with anti-human IgG AP-conjugated antibodies (diluted 1∶10,000; Promega). Proteins reacting with the antisera were visualized by use of the chromogenic substrates BCIP and NBT.

### Generation of antisera against candidate adhesins

Five 5-week old female BALB/c mice were immunized intraperitoneally with 100 µg (in 100 µl volume) of each recombinant MBP fusions to *L. interrogans* adhesins (LIC10508, LIC11574, LIC12341, LIC13411, and control β-galactosidase) with 100 µl Alum adjuvant (Thermo Scientific, Rockford, Il). The mice were boosted 3 times at 14 day intervals with the same amount of protein in Alum. At day 56, the mice were bled by cardiac puncture and sera were prepared from the collected blood. Sera obtained from control groups inoculated with PBS alone or PBS with Alum, showed no reactivity against any of the candidate adhesins or *L. interrogans* lysate.

### Cellular fractionation of *L. interrogans*

The cellular localization of *L. interrogans* proteins was determined using fractionation and phase separation assays adapted from [50]. *L. interrogans* was grown in 800 ml EMJH medium at 30°C until late log phase (∼10^8^–10^9^/ml). Bacteria were harvested by centrifugation for 30 min at 2,683 x g. Bacterial pellets were washed 3x with PBS pH 7.4 supplemented with 5 mM MgCl_2_ at 4°C, then resuspended in ice cold 10 mM Tris-HCl, 2 mM EDTA (TE), pH 7.4 supplemented with protease inhibitors 0.01 TIU/ml aprotinin, 1 mM benzamidine and 10 µM pepstatin. *L. interrogans* concentration was adjusted to 5×10^9^ bacteria/ml in a total volume of 15 ml. Ice-cold 10% TX-114 (Sigma-Aldrich) in TE supplemented with protease inhibitors was added to the bacteria to a final concentration of 1%. The solution was gently mixed and incubated at 4°C for 45 min on a rocker. Following incubation, the bacterial suspension was centrifuged for 15 min at 17,000 x g at 4°C. The supernatant was transferred to a sterile tube and CaCl_2_ was added to the supernatant to a final concentration of 20 mM. The supernatant fraction was kept at 4°C until use for phase separation steps. The pellet was resuspended in 1 ml of final sample buffer (FSB; 62.5 mM Tris-HCl pH 6.8; 10% glycerol; 2% SDS supplemented with aprotinin, benzamidine and pepstatin). This is the detergent-insoluble fraction containing the protoplasmic cylinder (PC).

Ice cold 10% TX-114 was added to the supernatant (detergent soluble fraction) to a final concentration of 2%. Phase separation was performed by warming the detergent-soluble supernatant to 37°C for 10 min followed by centrifugation for 10 min at 1,000 x g. Centrifugation allows the separation of the detergent-poor aqueous phase (AQ) found at top from the detergent-rich hydrophobic phase (DET) observed as an “oily droplet” at the bottom of the tube. The AQ fraction is made up mostly of periplasmic proteins, while outer membrane lipoproteins and transmembrane proteins are found in the DET phase. The DET and AQ were then washed four times in the following manner. The aqueous phase was chilled in an ice bath, and 10% TX-114 was added to a final concentration of 2%, followed by warming to 37°C, then centrifugation at 2,000 x g at RT. The detergent phase was chilled in an ice bath, diluted with nine parts TE buffer, warmed to 37°C, and centrifuged as described above. The AQ and DET were precipitated with acetone and resuspended in FSB. *L. interrogans* whole cell lysates (WCL) were prepared by harvesting cultures, washing twice with PBS-MgCl, resuspended in PBS supplemented with protease inhibitors aprotinin and benzamidine, followed by sonication.

*L. interrogans* WCL, PC, DET and AQ fractions were run on a 12.5 or 15% SDS-PAGE gel, loading equivalents of 5×10^8^ or 2.5×10^9^ bacteria per lane. One gel was silver-stained and a duplicate gel was transferred to a PVDF membrane and blocked with 5% milk, 3% goat serum (GS) in TBS for 1 hr at RT. Immunoblots were incubated with antisera against control periplasmic protein flagellin A1 (FlaA1) (1∶5,000) which would be attached to the inner membrane, cytoplasmic membrane lipoprotein LipL31 (1∶5,000), outer membrane proteins OmpL47 (1∶5,000), OmpL1 (1∶5,000), and LipL21 (1∶5,000), and candidate *L. interrogans* adhesins LIC10508 (1∶100), LIC11574 (1∶100), LIC12341 (1∶100), and LIC13411 (1∶500) in blocking buffer overnight at 4°C. After washing 3x with TBS, the membranes were probed with 1∶10,000 goat anti-rabbit antibody conjugated to AP or anti-mouse antibody conjugated to HRP for 40 min at RT. The membranes were washed with TBS 3x followed by visualization of protein bands using chromogenic substrates NBT and BCIP or by enhanced chemilumescent (ECL) substrate (Thermo Scientific).

### Statistical analysis

Analyses were performed using Prism 6 (GraphPad Software, Inc) utilizing two-tailed Student's *t-*test. *P* values lower than 0.05 were considered statistically significant.

## Results

### Identification and characterization of phage clones that bind mammalian host cells

To identify bacterial cell surface proteins that mediate attachment to host cells, a *L. interrogans* filamentous phage display library was constructed. The vector used in generating the library, fdBUG, cannot express the phage coat protein III because of the stop codons in all three reading frames near the 5′ end of gene III [37]. Cloning of *L. interrogans* DNA fragments that encode an open reading frame continuous with that of the phage gene III allows expression of leptospiral peptides as fusions to the minor coat protein on the surface of the phage. Based on the average *L. interrogans* DNA fragment size of 500 bp (inserted in fdBUG vector) and its genome size of 4.5 Mbp, the number of clones necessary to represent the entire genome is ∼41,800. The number of transformants recovered was 1.2×10^6^, with approximately 82% of the clones containing *L. interrogans* DNA inserted in the phage vector. It should be noted that the leptospiral DNA inserted in the phage vector was ligated with 3 pairs of adaptors, so one of the nine possible combinations will result in production of the protein encoded by the inserted DNA in frame with phage protein III. Therefore, the total number of suitable clones obtained is 1.1×10^5^, enough to represent the genome with approximately 2.6-fold redundancy.

To select for phage clones that bind specifically to cells, five independent pools of phage were pre-adsorbed on ECM and the unbound phage particles were subjected to three rounds of selection for phage that bind to EA.hy926 endothelial cells *in vitro*. The endothelial cell line EA.hy926 was used in this study because it represents a cell type that is relevant to *Leptospira* infection and because of its relative ease in cultivation. Additionally, *L. interrogans* exhibited attachment to EA.hy926 monolayers *in vitro* [8], [11], [35], and binding to the cells was stronger than to the ECM [35]. As a control, a parallel selection was also performed using fdDOG to assess non-specific binding of phage to cells. The number of clones eluted after each round of selection against endothelial cells increased as selection progressed. After the third round of selection, the number of colonies recovered from the phage library was on average 70-fold higher compared to the control fdDOG.

**Figure 1:**
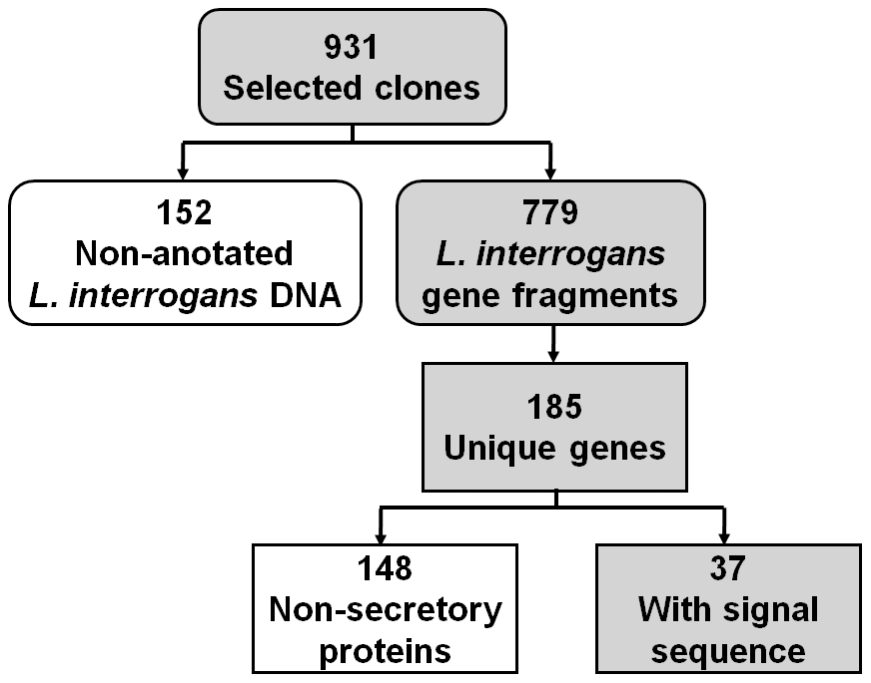
Schematic of analysis of phage clones that bind to endothelial EA.hy926 monolayers. Following three rounds of selection for phage clones that bind endothelial cells, 931 clones were sequenced and analyzed by BLAST. Seven hundred seventy-nine phage clones have DNA inserts of *L. interrogans* open reading frames, corresponding to 185 unique genes. Using prediction programs SignalP 3.0 and LipoP 1.0, 37 of the 185 identified genes contain signal peptide at the N-terminus of the predicted protein product.

A total of 931 clones from the three rounds of selection for binding to endothelial cells were sequenced (Figure 1). To identify the *L. interrogans* gene fragments inserted in the selected phage, the sequences obtained were compared by BLAST searches with the complete genome of *L. interrogans* serovar Copenhageni strain Fiocruz L1–130. Out of the 931 sequences analyzed, 152 clones have inserts of *L. interrogans* DNA outside of annotated genes while 779 clones have DNA inserts corresponding to open reading frames, representing 185 unique genes (Figure 1). Many of the top gene hits in the selection encode proteins that do not possess a signal peptide (Table S1). Because the proteins encoded by these genes are not predicted to be present on the bacterial cell surface and there is no literature suggesting their surface localization, these gene hits were not further pursued. Although the number of hits may suggest strong selection to bind to endothelial cells, these interactions may not be biologically relevant if these proteins are not found on the surface of the bacterial cell. A similar phenomenon was previously observed using this system [37].

Since our aim was to identify bacterial proteins that interact with the host, *L. interrogans* genes encoding proteins that are predicted to be surface-localized or found extracellularly were of interest and priority. The gene hits were analyzed using programs SignalP 3.0 and LipoP 1.0 for the presence of a signal peptide at the N-terminus of the predicted protein product. All spirochetal OMPs studied have signal peptides that initiate export of the protein and are subsequently cleaved following translocation [47]. Out of the 185 genes analyzed, 37 were predicted to encode proteins that contain secretion signal sequences (Table S2), and therefore at least may be exported out of the cytoplasm. Four genes were prioritized for further screening based on additional criteria described in the Materials and Methods. These include *L. interrogans* genes predicted to express putative lipoproteins LIC10508 and LIC13411, and conserved hypothetical proteins LIC11574 and LIC12341 (Table 2).

**Table 2:** *L. interrogans* genes encoding known or putative surface proteins selected for binding to endothelial cells.

Gene	Protein	SignalP3.0/ LipL1.0	*Lsp or Lep cleavage	PRED-TMBB	Predicted Protein Size (kDa)	Strains in which the gene is found	Known or putative surface localization as described in literature
LIC10508	Putative lipoprotein	+	Lsp	No β-barrel motif	21.0	Pathogen	[65]
LIC11574	Conserved hypothetical protein	+	Lep	2 β-barrel motifs	18.1	Saprophyte, pathogen	[65]
LIC12341	Conserved hypothetical protein	+	Lep	8 β-barrel motifs	21.2	Pathogen	-
LIC13411	Putative lipoprotein	+	Lsp	No β-barrel motif	24.6	Saprophyte, pathogen	-

Phage clones pre-adsorbed on ECM and subjected to three rounds of selection in EA.hy926 cells were analyzed to identify the inserted *L. interrogans* sv. Copenhageni st. Fiocruz L1–130 gene. Out of the 931 phage clones selected, 779 have *L. interrogans* DNA inserts that represent 185 unique genes. Thirty-seven of these genes contain signal sequence. Four genes shown below were prioritized for further assessment.

*During translocation, the signal sequence of lipoproteins and transmembrane OMPs are cleaved by lipoprotein signal transpeptidase (Lsp) and leader peptidase (Lep), respectively.

Alignment of the *L. interrogans* peptides encoded in the selected phage clones revealed diverse sequences without any obvious consensus (Table S3). This is not surprising given that we selected phage clones using intact cells. It should be noted that each cell type expresses a number of different receptors that can potentially interact with clones in a phage display library [8], [35]. This accounts for why we obtained a number of peptides with no clear consensus.

**Figure 2:**
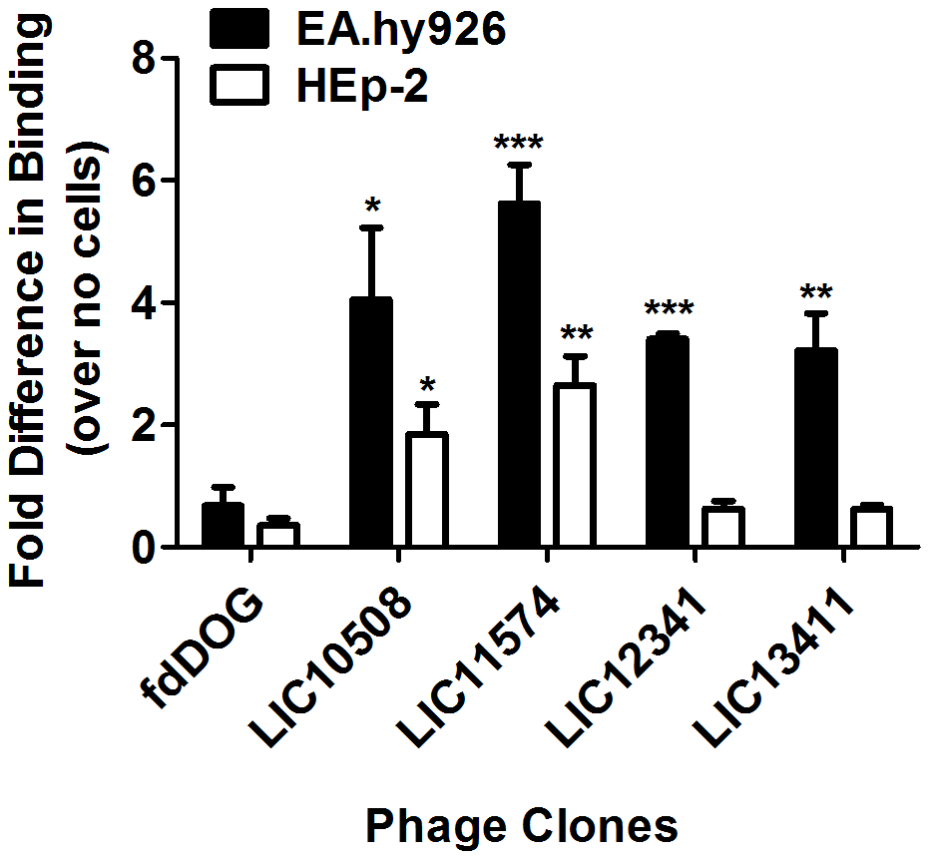
Selected phage clones bind to endothelial and epithelial cells. Individual phage clones (∼10^10^) were added with confluent endothelial EA.hy926 or epithelial HEp-2 monolayers, or to wells containing cell medium only for 2 hr at 37°C under 5% CO_2_. Phage attachment was quantified by ELISA using an anti-M13 (fd phage coat) antibody. Vector phage clone fdDOG [37] serves as a negative control. Binding is expressed as fold difference in the absorbance between monolayers vs. wells containing medium alone. Results are expressed as means ± standard errors, representative of at least two independent experiments repeated in quadruplicate. Error bars represent standard error while asterisks (*) indicate significant difference in attachment of candidate adhesins to cells compared to fdDOG (two-tailed *t-*test, *P<0.05, **P<0.01, ***P<0.001).

To confirm cell-specific attachment of the selected phage clones, purified phage particles were prepared from individual phage clones and were tested for binding to cell monolayers. Phage suspensions were incubated with confluent monolayers of EA.hy926 or HEp-2 cells. The unbound phages were washed off and the phages bound to cells were fixed and quantified in an ELISA-based assay using anti-M13 (fd phage coat) antibodies. Cell monolayers were also incubated with fdDOG as control. Results were expressed as fold difference in the absorbance readings in the presence of cells over no cells. All phage clones carrying *L. interrogans* gene fragments showed binding to EA.hy926 endothelial cells (≥4 fold compared to vector control) (Figure 2). Phage clones expressing fragments of the LIC10508 and LIC11574 genes also adhered to the epithelial cell line HEp-2. Collectively, the selected phage clones bind more efficiently to endothelial as compared to epithelial monolayers, demonstrating that the selection process was successful in identifying clones that bind directly to this endothelial cell line. The observed disparity in the binding of phage clones between the two cell lines can be attributed to the likelihood that the receptors recognized by these phage clones are differentially expressed by the cell lines used.

### Attachment of recombinant *L. interrogans* candidate adhesins to cell monolayers

To determine whether the *L. interrogans* proteins encoded by genes selected through phage display bind to host cells beyond the context of the phage particle, recombinant forms of LIC10508, LIC11574, LIC12341 and LIC13411 were generated. The genes were cloned in pMalC2 expression vector to generate the mature protein (without the signal peptide) fused to maltose binding protein (MBP) (Figure S1). The 43 kDa MBP was chosen as tag as it increases the solubility of many fusion proteins, and the recombinant proteins can easily be purified by amylose affinity chromatography. Recombinant forms of other known adhesins such as invasin from *Y. pseudotuberculosis* and P66 of *Borrelia burgdorferi* have been successfully generated using this vector [37], [48], [51]. The relative binding capacities of the MBP fusions to endothelial and epithelial cells were determined using an ELISA format. For the binding assays, we chose to use the microvascular endothelial cell line HMEC-1 because of the physiological relevance of this cell type in the widespread hemorrhage observed in patients with leptospirosis.

**Figure 3:**
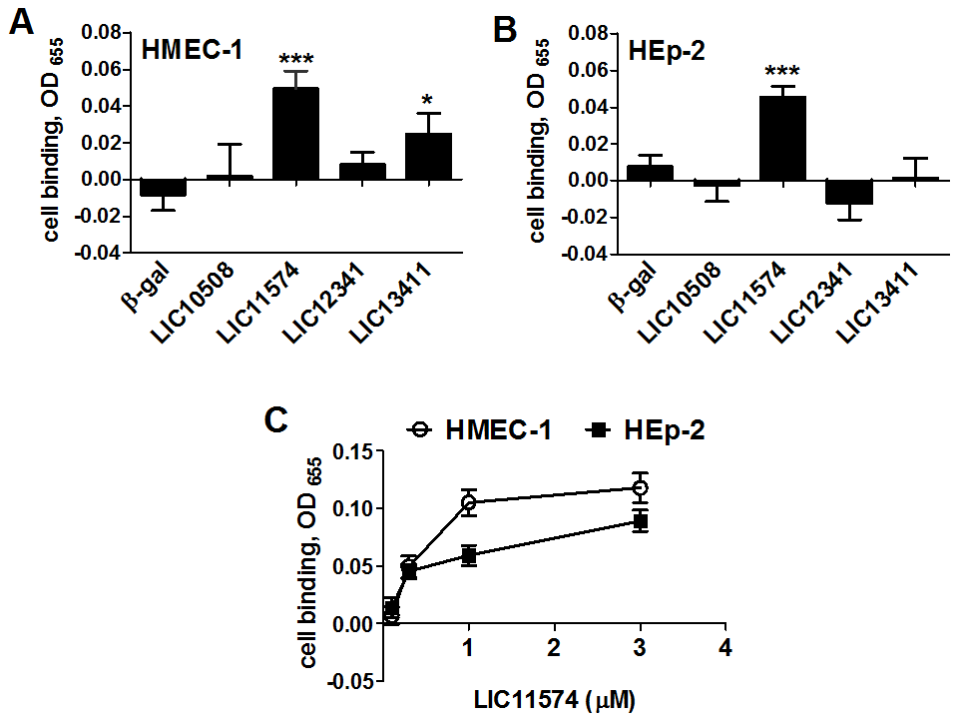
Recombinant *L. interrogans* candidate adhesins bind to cells. Recombinant forms of *L. interrogans* candidate adhesins expressed as fusions to MBP, along with β-galactosidase (β-gal) were diluted to 0.3 µM and incubated with confluent monolayers of endothelial HMEC-1 (Panel A) or epithelial HEp-2 (Panel B) cells for 1 hr at 37°C, 5% CO_2_. The unbound proteins were removed by washing and the bound adhesins were detected by ELISA using anti-MBP antibody. Results were expressed as the difference in the absorbance reading between wells with or without cells. The data shown are means ± standard errors from 8 independent assays with n = 8–28. Asterisks (*) indicate significant difference in binding when compared to the MBP fusion to β-gal, the negative control (two-tailed *t-*test, *P<0.05, ***P<0.001). Panel C shows concentration-dependent attachment of LIC11574 to endothelial and epithelial cells.

Increasing concentrations of recombinant proteins were incubated with confluent cell monolayers for 1 hr. The protein bound to cells was quantified and expressed as the absorbance reading of proteins in wells containing cells minus background signal or plastic binding. MBP-β-galactosidase and MBP-invasin served as negative and positive controls, respectively. The recombinant form of the conserved hypothetical protein LIC11574 demonstrated binding to HMEC-1 cell monolayers in a dose-dependent and saturable manner. The putative lipoprotein LIC13411 also binds HMEC-1 cells (Figure 3A). Attachment of LIC11574 to epithelial HEp-2 cell monolayers was significantly higher as compared to control protein (Figure 3B), but was not saturable (Figure 3C). Both endothelial and epithelial cells bound the control protein, invasin efficiently (not shown).

**Figure 4:**
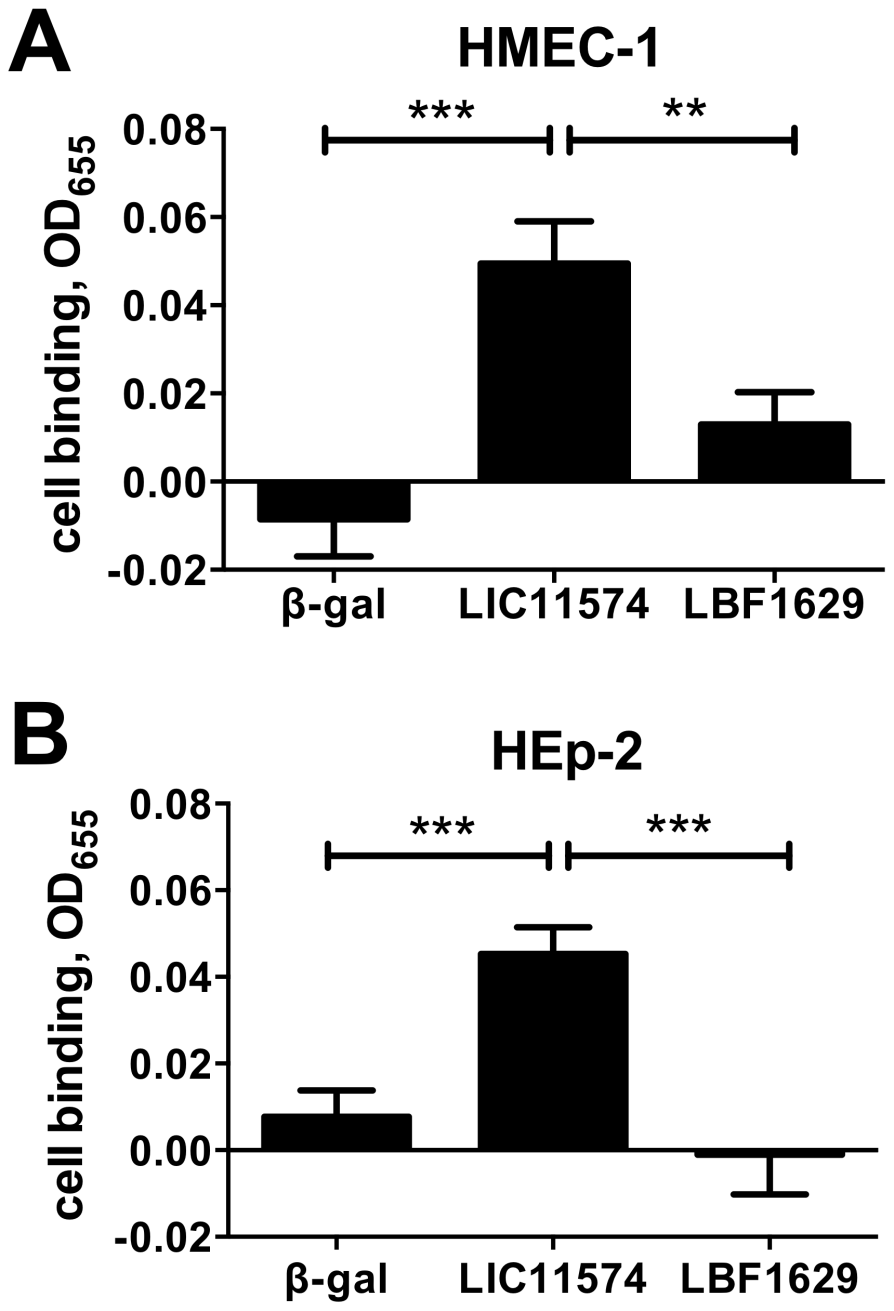
*L. interrogans* candidate adhesin LIC11574, but not its *L. biflexa* ortholog, LBF1629, binds to cells. MBP fusions of LIC11574 and LBF1629, along with control β-galactosidase (β-gal) were diluted to 0.3 µM and incubated with confluent monolayers of HMEC-1 endothelial (Panel A) or HEp-2 epithelial (Panel B) cells for 1 hr at 37°C, 5% CO_2_. The unbound proteins were removed by washing and the bound adhesins were detected by ELISA using anti-MBP antibody. Results are expressed as the difference in the absorbance reading between wells with or without cells. The data shown are means ± standard errors from 8 independent assays with n = 16–28. Asterisks (*) indicate significant difference in binding when compared to the MBP fusion to β-gal, the negative control (two-tailed *t-*test, *P<0.05, ***P<0.001). Results show that LBF1629 (the *L. biflexa* ortholog of LIC11574) does not have cell-binding activity.

LIC11574 has a homologue in the saprophytic *L. biflexa* sv. Patoc st. Patoc I (Ames) annotated as LBF1629 (Accession number YP_001962715). The 24.3 kDa protein product is annotated as conserved hypothetical protein containing a possible Type IV pilin N-terminal methylation site [52]. LBF1629 has a 33% identity and 55% similarity to LIC11574. To assess the cell-binding activity of the protein encoded by the saprophytic strain, a recombinant form of LBF1629 was tested in adhesion assays and its binding activity was compared to LIC11574. Results show that LBF1629 did not bind to either HMEC-1 endothelial or HEp-2 epithelial cells after 1 hr of incubation (Figure 4). This may suggest that despite some similarity in the amino acid sequences of these two proteins, there is a difference in the function of the proteins encoded by the pathogenic and saprophytic strains.

### Binding of candidate adhesins to cadherins

Our group has previously identified the cadherins as receptors for *L. interrogans* [35]. To determine whether the candidate adhesins facilitate bacterial attachment to vascular endothelial (VE)-cadherin, we incubated MBP fusions of LIC10508, LIC11574, LIC12341, and LIC13411 with immobilized VE-cadherin. The bound adhesins were quantified using the ELISA approach described above.

**Figure 5:**
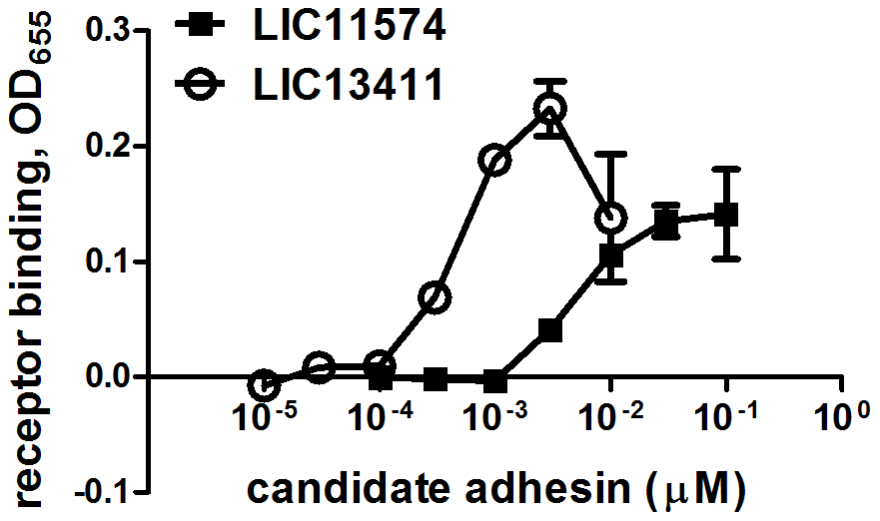
*L. interrogans* candidate adhesins LIC11574 and LIC13411 bind to the host protein VE-cadherin. *L. interrogans* proteins, LIC11574 and LIC13411 were expressed as fusions to MBP (diluted 1×10^−5^–0.1 µM) and incubated for 1 hr with 0.01 µM VE-cadherin or control BSA immobilized on a Linbro plate. The unbound recombinant proteins were washed off and bound proteins were quantified by ELISA using an anti-MBP antibody. Attachment to VE-cadherin is expressed as the OD reading at 655 nm minus BSA and no receptor (or “plastic”) binding. Results indicate that attachment of LIC11574 (*K*_D_ = 7.6±2.5 nM) and LIC13411 (*K*_D_ = 0.4±0.2 nM) to VE-cadherin, but not to BSA, is dose-dependent and saturable.

Both LIC11574 and LIC13411 exhibited higher adherence to VE-cadherin than did the negative control β-gal. Interestingly, both of these proteins also exhibited attachment to HMEC-1 cells, which have been previously shown to express VE-cadherin [35]. This result suggests possible roles of these adhesins in mediating bacterial attachment to endothelial cells through the cadherin receptors. A dose response assay was performed to quantify the binding of LIC11574 and LIC13411 to VE-cadherin vs. control BSA immobilized on wells (Figure 5). The results show that LIC11574 binds efficiently to VE-cadherin with an estimated *K*_D_ = 7.6±2.5 nM. The binding of LIC13411 to VE-cadherin was also efficient, *K*_D_ = 0.4±0.2 nM. The binding of both adhesins to VE-cadherin was dose-dependent and saturable. LIC13411 also showed binding to BSA at high recombinant protein concentrations, but this was not saturable.

### Recognition of recombinant proteins by sera from leptospirosis patients

To determine if our candidate adhesins are expressed during *Leptospira* infection, we screened sera from confirmed leptospirosis patients in the convalescent phase and from control individuals for reactivity to the adhesin proteins by immunoblot analysis. For this assay, sera were initially pre-cleared by incubation with membranes blotted with whole cell lysate of *E. coli* expressing MBP-β-galactosidase. MBP is expressed by *E. coli*, which is part of the gut flora of both humans and animals [53]. This step is to ensure that anti-MBP- or other anti-*E. coli-*specific antibodies are removed from the sera, and thus reduces non-specific signals.

None of the sera from healthy individuals recognize recombinant forms of LIC12341, LIC13411, LIC11574 or LIC10508 (Table 3). In contrast, all candidate adhesins were recognized by at least one of the five sera from patients with leptospirosis. All patient sera recognized OmpL1, a known OMP [54], [55] that was used as the positive control for the test. β-galactosidase was used as negative control and did not react with any of the pre-cleared sera tested.

**Table 3:** Expression of candidate adhesins during *Leptospira* infection.

Candidate adhesins	Leptospirosis Patient Sera	Control Sera
LIC10508	4/5	0/5
LIC11574	3/5	0/5
LIC12341	4/5	0/5
LIC13411	1/5	0/5
β-galactosidase	0/5	0/5
OmpL1	5/5	0/5

Six hundred nanograms of MBP fusions to *L. interrogans* candidate adhesins were immobilized on membrane and probed with pre-cleared convalescent sera from patients diagnosed with leptospirosis or control individuals by western blot. Sera were used at 1∶500 dilution.

### Cellular localization of candidate adhesins

Cellular fractionation of *L. interrogans* using Triton X-114 followed by phase partitioning experiments was performed to determine cellular localization of candidate cell-binding adhesins. This non-ionic detergent allows solubilization of the outer membrane but keeps the cytoplasmic membrane intact. Shifting the temperature from 4°C to 37°C allows separation of hydrophilic and hydrophobic fractions. TX-114 has been extensively used in the study of spirochete OMPs [32], [50], [56]–[60]. *L. interrogans* Copenhageni was treated with TX-114 followed by centrifugation to separate the detergent-insoluble material or protoplasmic cylinder fraction (PC), made up of the inner membrane, bacterial components attached to the inner membrane, and cytoplasmic contents. Phase separation was performed by warming the detergent-soluble supernatant to 37°C followed by centrifugation to separate the detergent-rich hydrophobic phase (DET) from the detergent-poor aqueous phase (AQ). The AQ fraction is made up mostly of periplasmic proteins while outer membrane lipoproteins and transmembrane proteins are found in the DET phase. As a control, whole cell lysates (WCL) of leptospires were prepared by sonication. Different fractions were assessed by western blot using antisera against *L. interrogans* proteins with known cellular locations such as inner membrane-associated FlaA1 and LipL31, and outer membrane proteins OmpL47 and OmpL1.

**Figure 6:**
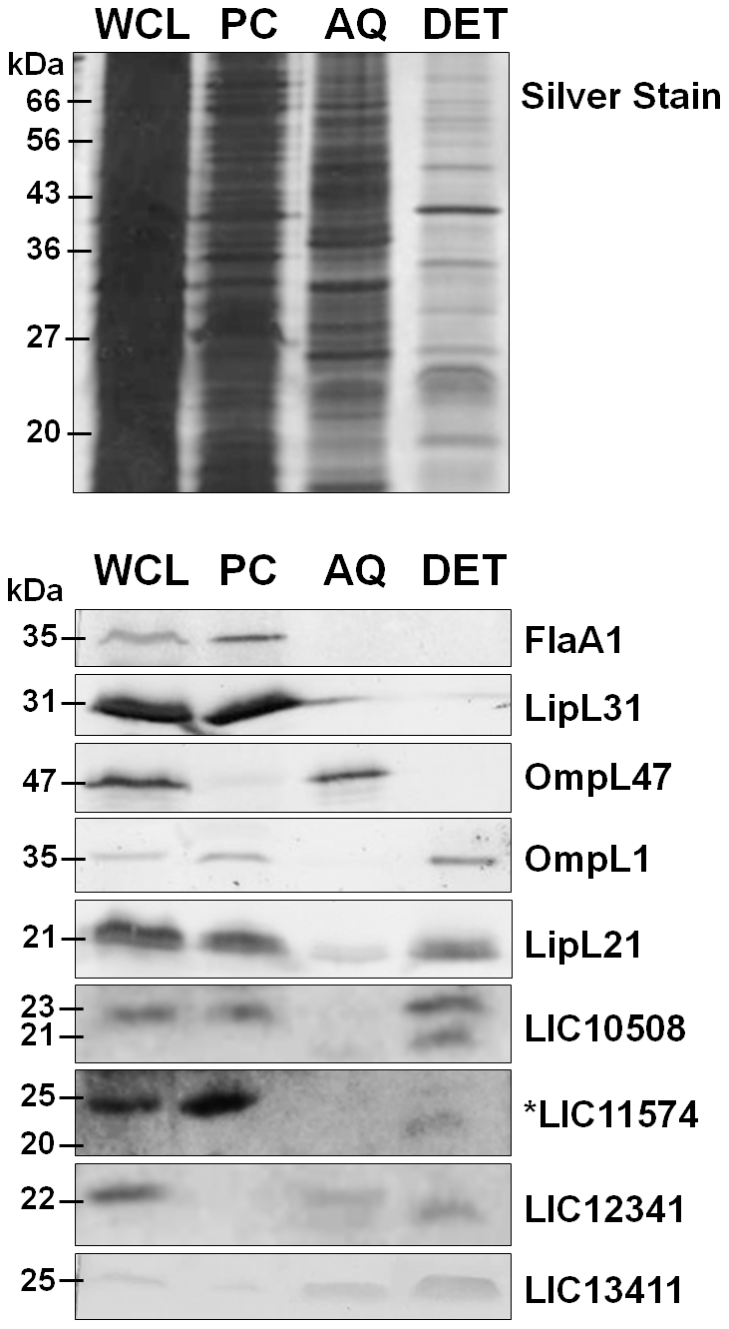
Localization of proteins after Triton X-114 fractionation of *L. interrogans* sv. Copenhageni. *L. interrogans* was phase partitioned using Triton X-114 as described in Materials and Methods. Equivalents of 5×10^8^ or 2.5×10^9^ (*) spirochetes/lane of the whole cell lysate (WCL), protoplasmic cylinder (PC), aqueous (AQ) and detergent (DET) fractions were separated on 12.5 or 15% SDS-PAGE electrophoresis, transferred to PVDF membrane and probed with rabbit immune sera against periplasmic protein flagellin A1 (FlaA1), inner membrane protein LipL31, outer membrane proteins OmpL47 and OmpL1, and mouse immune sera against candidate adhesins LIC10508, LIC11574, LIC12341, and LIC13411. A duplicate gel was silver stained.

The TX-114 fractionation analysis (Figure 6) showed that the periplasmic protein FlaA1 and inner membrane protein LipL31 remain in the protoplasmic cylinder fraction (PC), as expected. The transmembrane OMP OmpL47 was solubilized by the detergent but fractionates in the AQ phase. A similar result was observed by Pinne and Haake [50] which was unanticipated by the authors. However, additional assays such as surface proteolysis, immunofluorescence, membrane affinity and surface biotinylation confirmed that OmpL47 is indeed surface-exposed and membrane-integrated. OmpL1 is another transmembrane protein but behaves differently from OmpL47. Both OMPs LipL21 and OmpL1 are partially solubilized by TX-114 extraction but partition exclusively to the DET phase [32].

All candidate adhesins were observed in the DET fraction. LIC10508 and LIC11574 behaved similarly to LipL21 and OmpL1, with both proteins also detected in the protoplasmic cylinder after TX-114 treatment. LIC10508 has previously been reported to fractionate in the detergent phase, and to induce production of ICAM-1 and E-selectin by HUVEC cells, although direct adhesion to the cells or to extracellular matrix molecules was not demonstrated [61]. LIC10508 migrated at 23 kDa, slightly larger than the predicted 21 kDa mature protein product. Interestingly, two protein bands for LIC10508, 21 kDa and 23 kDa were observed at the DET phase. LIC11574 also showed two different-sized species in different fractions; a band in detergent migrated faster (20 kDa) than bands in detergent-insoluble fraction and in the whole cell lysate (25 kDa). Similarly, LIC12341 also showed two distinct species in different fractions. The different apparent molecular weights of LIC10508, LIC11574, and LIC12341 in different fractions, with the lower apparent M_r_ forms appearing in the detergent-soluble phase, suggests that the proteins may be undergoing processing. While differences in M_r_ between the protoplasmic cylinder fraction, which might include protein molecules that have not yet had secretion signal sequences removed, and the outer membrane fraction would not be surprising, the differences seen between the aqueous and detergent-soluble portions of the outer membrane fraction suggest that additional post-translational modifications might occur for these proteins. LIC11574 was observed in the AQ fraction when we increased the bacterial load to equivalents of 5×10^9^ per lane. LIC12341 and LIC13411 were both found to be completely TX-114 soluble but found in DET and AQ phases. Lower dilutions of the antisera against the cell-binding adhesins described here (1∶100 or 1∶500) were used to detect the *L. interrogans* candidate adhesins, in comparison to the sera used to detect other proteins used as markers of cellular fractionation. This low reactivity is likely due to the low abundance of proteins expressed by *L. interrogans* because all antisera recognized the recombinant candidate adhesins at 1∶20,000 dilution; this is consistent with low transcript abundance for these adhesins [62].

## Discussion

The binding of *Leptospira* spp. to ECM components of the host has long been established [17], [20], [21], [23]–[25], [30], [34]. However, our group has shown that *L. interrogans* binds more efficiently to cells than to ECM *in vitro* [8], [35], and that the host cell surface protein VE-cadherin contributes to bacterial attachment to endothelial cells [35]. With the limitations in the available genetic tools in *Leptospira* spp., phage display represents an alternative but powerful tool to identify *Leptospira* proteins with cell-binding activities, as well as the specific portions of the proteins with adhesin activities. This approach has also been employed in identifying *Leptospira* proteins that bind to ECM components [63]. Our experimental strategy eliminated adhesins that might bind to host molecules that might be deposited as extracellular matrix, or remain cell-associated. Our focus, however, was on cell-surface receptors due to the potential for trans-membrane signaling that could allow the bacteria to manipulate host cell biology to their own advantage.

The putative lipoproteins LIC10508 and LIC13411, and conserved hypothetical proteins LIC12341 and LIC11574 were identified using phage display and were prioritized for further screening. Studies focusing on these four candidate adhesins are sparse in the literature. Pinne *et al.* [64] described all four proteins as probable OMPs using a set of algorithms and criteria to identify candidate lipoproteins and transmembrane OMPs in *L. interrogans* sv. Copenhageni st. Fiocruz L1–130. Using a protein microarray screening assay, LIC13411, LIC11574 and LIC10508 all exhibited low binding activities to fibronectin [64]. Gamberini and colleagues also identified LIC10508 (putative lipoprotein; LipL23) and LIC11574 (conserved hypothetical protein; OmpL22) as surface proteins using *in silico* analysis [65]. Both proteins reacted with *Leptospira*-infected human sera suggesting that these proteins are expressed in the course of human infection. A serological cross reactivity assay performed by the group also demonstrated that LIC10508 is highly conserved among pathogenic strains. The cell-binding properties of these proteins are first described in this work.

One advantage of phage display is that it allows the mapping of the binding domains, providing information on the peptide sequence sufficient to establish interactions with the substrate. In this study, the alignment of the *L. interrogans* peptide fragments inserted in the cell-binding phage clones did not reveal any discernable pattern. This was not surprising as the selection of the phage library was carried out using intact monolayers rather than a homogenous substrate, suggesting that the different selected phages may have recognized and bound various components of the monolayer. It should be noted that this study started prior to the identification of cadherins and other proteins as *L. interrogans* receptors on host cell surfaces [35]. In addition, our finding that phage clones carrying *L. interrogans* gene fragments bind more efficiently to endothelial cells than epithelial monolayers was not surprising since selection was carried out using an endothelial cell line. This disparity in the binding activities may be attributed to differences in the level or type of receptors expressed between the two cell types.

We speculate that *L interrogans* adhesins recognize and bind specific receptors that are differentially expressed in different cell types. We have previously demonstrated that the production of host receptor proteins VE-cadherin, E-cadherin and ICAM-2 varies among the various endothelial and epithelial cell lines tested [35]. This finding suggests that pathogenic *Leptospira* spp. have evolved mechanisms to adhere to a variety of cell types through expression of numerous adhesins. The attachment of *L. interrogans* to host molecules is thought to be important to establish bacterial infection and dissemination resulting in leptospirosis.

Like any experimental approach, the use of phage display has its caveats. One limitation of this approach is that peptides displayed on the surface of the phage may be folded differently from the purified recombinant fusion forms or on the surface of the leptospires (*in vivo*). Phage display is limited to protein fragments that some host cell receptors may not bind if three-dimensional structures are required. This may explain the differences in the binding activities of the phage clones expressing adhesin peptide versus purified recombinant forms of the intact candidate adhesins. Both LIC11574 and LIC13411 displayed high affinity binding to VE-cadherin, a recently identified host protein receptor for *L. interrogans* [35]. However, whether the abilities of LIC11574 and LIC13411 to bind to purified VE-cadherin contribute to the ability of pathogenic *Leptospira* spp. to attach to endothelial cells, and whether multivalent interactions are required, need to be further explored. The interactions of pathogenic leptospires with VE-cadherin, in particular, is of interest due to the critical importance of VE-cadherin in maintenance of endothelial integrity [66], [67] and the disruption thereof by pathogenic *Leptospira* species. The roles of LIC11574 and LIC13411 in pathogenesis as potential virulence factors also need to be established, and the potential for functional redundancy requires further exploration. Generation of targeted knockout mutants in each of these genes will be required to address these questions, and it is possible that inactivation of both genes will be required to detect a phenotype *in vivo*.

It is thought that the ability of the pathogenic *Leptospira* spp. to recognize and bind host surface components is an essential step in the pathogenesis of leptospirosis. Outer membrane proteins are likely mediators of these interactions, leading to the entry and dissemination of the pathogen. This has led to several studies identifying OMPs and evaluating their roles in virulence. OMPs that are unique to pathogenic strains are of interest to a number of investigators, as their presence exclusively in infectious strains may support their role in virulence. One of our candidate adhesins, LIC11574, has a homologue in the saprophytic strain *L. biflexa*. Only the OMP encoded by the pathogen demonstrated binding to cell monolayers. This suggests either there are distinct functions between protein homologues or adhesion is an additional role acquired during evolution in pathogenic strains to survive in hosts. Most studies focus on the roles of proteins unique to infectious strains but proteins shared by all *Leptospira* species should not be overlooked. Our findings may suggest that small differences in primary, secondary, or tertiary structures may bring about significant disparities in the roles of the proteins.

Development of classic genetic knock-outs to assess roles in virulence may not be ideal in this system because of the potential for redundancy in the functions of adhesins. Loss of a gene (inactivation) may not result in obvious phenotypic change if the function is redundant to that of another protein with the same activity. For example, site-directed mutagenesis through allelic exchange was successful in the inactivation of *ligB* [68], but the loss of this ECM-binding adhesin did not affect the ability of the pathogenic *L. interrogans* to infect hamsters. The expression of pathogen-specific LigB on saprophytic *L. biflexa* resulted in enhanced bacterial attachment to ECM and cells in culture [24]. Therefore, gain-of-function approaches by heterologous expression of adhesins from pathogens in saprophytic *L. biflexa* at least allowed confirmation of the role of this protein in adhesion to host cells *in vitro*. Interestingly, we recovered a fragment of LigB that did not contain any of the known ECM-binding domains [28], [31] in our selection using endothelial cells (Table S2). This result will be pursued in the future.

The mechanism of host resistance to pathogenic *Leptospira* spp. is mediated largely by humoral responses [7], [69], [70]. The candidate adhesins identified by phage display, LIC12341, LIC13411, LIC11574 and LIC10508, are recognized by sera from convalescent leptospirosis patients but not by sera from control individuals. This implies expression of these proteins during infection. Because of their accessibility to host immune responses during infection, a number of surface-exposed *Leptospira* OMPs are considered as candidate vaccinogens. Leptospiral OMPs OmpL1 and LipL41 were protective immunogens when used in combination [71], which is promising in the development of subunit vaccines with greater protective effects and fewer side effects. Further tests are necessary to determine if the adhesins identified in this work have potential immunoprotective properties.

Bacterial adhesive molecules have surface-exposed moieties that allow them to interact with host cells, and may play important roles in establishing bacterial attachment and dissemination in hosts. Therefore, determining surface localization is vital to support the role of pathogenic *Leptospira* OMPs in virulence. *In silico* approaches can help predict protein export from cytoplasm to periplasm [47] and lipidation of lipoproteins [72] however, direct examination is necessary to validate their cellular localization. We used a non-ionic detergent Triton X-114 as other groups were successful in removing spirochete outer membrane using this detergent [50], [73].

Our data reveal that all candidate proteins are found in the DET phase suggesting possible surface localization. Although the majority of LIC10508 and LIC11574 partition in the DET, these adhesins were also observed in the protoplasmic cylinder. Similar results were observed with the transmembrane porin OmpL1 [32], LipL21 [58], and other known *Leptospira* OMPs. Several groups have suggested that the incomplete detergent solubilization of these proteins may be attributed to amphipathic properties of transmembrane OMPs [32], [58]. The candidate adhesins LIC12341 and LIC13411 were completely solubilized in TX-114 but fractionated to both AQ and DET. Although the results were unexpected, similar findings were observed with the identified outer membrane protein OmpL54 [50]. We also observed low expression of the candidate adhesins, which corresponds to the paucity of their transcript levels [62]. This precluded the use other approaches such as immunofluorescence and surface proteolysis. However, multiple approaches to determining whether spirochetal proteins are localized on the outer surface of the bacterial cell, as outlined by Pinne and Haake [50], [74], will be prioritized in further studies of our candidate adhesins.

Prior to this work, the attachment of pathogenic *L. interrogans* to host molecules was mainly attributed to the ability of bacterial OMPs to recognize and bind to ECM components. The study presented here provides evidence of direct binding of *L. interrogans* to host cells, and identifies bacterial components potentially involved in this interaction. Identification of molecular components necessary for the initial interaction between host and pathogen will greatly improve our understanding of *Leptospira* spp. pathogenesis. Surface-exposed OMPs that are recognized by the host immune response early during infection and play a role in bacterial virulence are ideal targets for diagnostic screening and development of vaccines. With the current robust leptospiral research output, in the near future we may see the development of simple and inexpensive diagnostic systems appropriate for highly endemic, resource-poor areas, as well as the application of state-of-the-art technologies to vaccine development.

## Supporting Information

Figure S1 **Purified recombinant MBP fusions to** ***L. interrogans*** **proteins.** *L. interrogans* proteins were expressed as fusions to maltose-binding protein (MBP) and purified by amylose affinity chromatography. Induction and expression of the pMalC2 vector alone yields MBP-β-galactosidase (β-gal). Three hundred ng of each MBP fusion were run on 12.5% SDS-PAGE gel and stained with Coomassie.
(TIFF)

Table S1 ***L. interrogans*** **genes inserted in selected phage clones with multiple hits.** Phage clones after three rounds of selection in EA.hy926 cells were analyzed to identify the inserted *L. interrogans* serovar Copenhageni strain Fiocruz L1–130 gene. Out of the 931 phage clones selected, 779 phage clones have *L. interrogans* gene fragment inserts representing 185 unique genes. Shown below are the 10 *Leptospira interrogans* genes with the highest number of hits after three rounds of selection. None of the proteins encoded by these genes contain signal peptides as determined by the prediction programs SignalP 3.0 or LipoP 1.0. However, LIC11570 (*) was previously described as an OMP [75] and manual analysis of its amino acid sequence indicate the presence of a Lep-recognized signal peptide.
(DOCX)

Table S2 **Selected** ***L. interrogans*** **genes encoding proteins with signal sequences.** Out of the 931 phage clones selected, 779 have *Leptospira* DNA inserts that represent 185 unique genes. Thirty-seven of these genes encode proteins containing signal sequence and are shown below. Those appearing in bold were prioritized for further characterization. * Known *L. interrogans adhesin* [20], [22], [24], [28], [30]–[31], [63]. ^a^ Inserted *L. interrogans* DNA in opposite orientation of the gene. ^b^ Inserted *L. interrogans* DNA in the N-terminal signal sequence.
(DOCX)

Table S3 **The amino acid sequences encoded in the DNA fragments inserted in the selected phage clones.** Alignment of the peptides did not reveal a common motif.
(DOCX)
