# Supplementary figures and images for: Identification of Cell-Binding Adhesins of Leptospira interrogans
Source: PLoS Negl Trop Dis. 2014 Oct 2;8(10):e3215. doi: 10.1371/journal.pntd.0003215 (PMC4183468; doi:10.1371/journal.pntd.0003215)

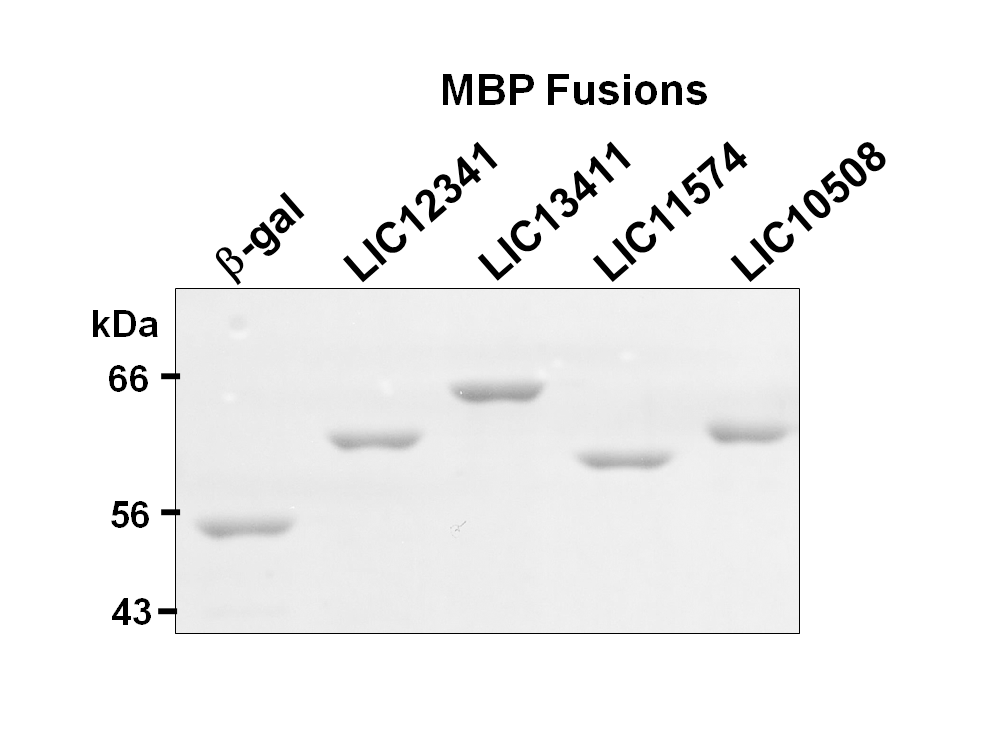

Supplement: Figure S1 — Purified recombinant MBP fusions to L. interrogans proteins. L. interrogans proteins were expressed as fusions to maltose-binding protein (MBP) and purified by amylose affinity chromatography. Induction and expression of the pMalC2 vector alone yields MBP-β-galactosidase (β-gal). Three hundred ng of each MBP fusion were run on 12.5% SDS-PAGE gel and stained with Coomassie. (TIFF) [file pntd.0003215.s001.tiff]
