# Supplementary material for: Identification of Cell-Binding Adhesins of Leptospira interrogans
Source: PLoS Negl Trop Dis. 2014 Oct 2;8(10):e3215. doi: 10.1371/journal.pntd.0003215 (PMC4183468; doi:10.1371/journal.pntd.0003215)
Supplement: Table S1 — L. interrogans genes inserted in selected phage clones with multiple hits. Phage clones after three rounds of selection in EA.hy926 cells were analyzed to identify the inserted L. interrogans serovar Copenhageni strain Fiocruz L1–130 gene. Out of the 931 phage clones selected, 779 phage clones have L. interrogans gene fragment inserts representing 185 unique genes. Shown below are the 10 Leptospira interrogans genes with the highest number of hits after three rounds of selection. None of the proteins encoded by these genes contain signal peptides as determined by the prediction programs SignalP 3.0 or LipoP 1.0. However, LIC11570 (*) was previously described as an OMP [75] and manual analysis of its amino acid sequence indicate the presence of a Lep-recognized signal peptide. (DOCX) [file pntd.0003215.s002.docx]

**Table S1. *L. interrogans* genes inserted in selected phage clones with multiple hits.**

| **Gene** | **Protein** | **# of Hits** |
| --- | --- | --- |
| LIC12400 | Isoleucyl-tRNA synthetase | 115 |
| LIC11081 | ABC transporter ATP-binding protein | 70 |
| LIC11483 | Conserved hypothetical protein | 68 |
| LIC20129 | Precorrin-2 C-20 methyltransferase | 53 |
| LIC11101 | Dihydroxy-acid dehydratase | 37 |
| LIC11756 | Nucleotide excision repair subunit C | 34 |
| LIC10502 | Cytoplasmic membrane protein | 23 |
| LIC11570* | General secretory pathway D | 16 |
| LIC10603 | Sulfatase family protein | 15 |
| LIC11417 | ATP-dependent Clp protease, proteolytic subunits | 14 |
