# Supplementary material for: Identification of Cell-Binding Adhesins of Leptospira interrogans
Source: PLoS Negl Trop Dis. 2014 Oct 2;8(10):e3215. doi: 10.1371/journal.pntd.0003215 (PMC4183468; doi:10.1371/journal.pntd.0003215)
Supplement: Table S2 — Selected L. interrogans genes encoding proteins with signal sequences. Out of the 931 phage clones selected, 779 have Leptospira DNA inserts that represent 185 unique genes. Thirty-seven of these genes encode proteins containing signal sequence and are shown below. Those appearing in bold were prioritized for further characterization. * Known L. interrogans adhesin [20], [22], [24], [28], [30]–[31], [63]. a Inserted L. interrogans DNA in opposite orientation of the gene. b Inserted L. interrogans DNA in the N-terminal signal sequence. (DOCX) [file pntd.0003215.s003.docx]

**Table S2. Selected *L. interrogans* genes encoding proteins with signal sequences.**

| **Gene Hits** | **# of Hits** | **Protein** |
| --- | --- | --- |
| LIC10044 | 1 | Conserved hypothetical protein |
| LIC10371 | 1 | Putative lipoprotein |
| LIC10432 | 1 | Cell division protein |
| LIC10464* | 1 | Ig-like repeat domain protein 3 (LigB) |
| **LIC10508** | 1 | Putative lipoprotein |
| LIC10544 | 1 | Outer membrane protein |
| LIC10590 | 1 | Conserved hypothetical protein |
| LIC10900 | 3 | Adenylate/guanylate cyclase |
| LIC10995 | 1 | Conserved hypothetical protein |
| LIC11036 | 1 | Conserved hypothetical protein |
| LIC11490 | 1 | Conserved hypothetical protein |
| LIC11505 | 1 | Conserved hypothetical protein |
| **LIC11574** | 2 | Conserved hypothetical protein |
| LIC11623 | 1 | Outer membrane protein |
| LIC11650 | 1 | Conserved hypothetical protein |
| LIC11738 | 1 | Penicillin binding protein |
| LIC11789 | 1 | Conserved hypothetical protein |
| LIC11864 | 1 | UDP-N-acetylglucosamine: LPS N-acetylglucosamine transferase |
| LIC11893 | 1 | Conserved hypothetical protein |
| LIC12067 | 1 | Hypothetical protein |
| LIC12285^a^ | 3 | H+-translocating pyrophosphatase |
| LIC12339 | 1 | Conserved hypothetical protein |
| **LIC12341** | 2 | Conserved hypothetical protein |
| LIC12342 | 1 | Export protein |
| LIC12525 | 1 | Putative lipoprotein |
| LIC12708 | 1 | Conserved hypothetical protein |
| LIC12988 | 1 | Lipase |
| LIC13001 | 1 | Conserved hypothetical protein |
| LIC13070 | 1 | Conserved hypothetical protein |
| LIC13076 | 1 | Putative lipoprotein |
| LIC13134 | 1 | Acriflavin resistance |
| LIC13341^b^ | 3 | Putative lipoprotein |
| LIC13361 | 1 | Conserved hypothetical protein |
| **LIC13411** | 2 | [Putative lipoprotein](http://www.ncbi.nlm.nih.gov/nucleotide/45602555?report=gbwithparts&from=4182149&to=4182937&RID=3DFA08RX01N) |
| LIC20054 | 1 | Conserved hypothetical protein |
