# Supplementary material for: Identification of Cell-Binding Adhesins of Leptospira interrogans
Source: PLoS Negl Trop Dis. 2014 Oct 2;8(10):e3215. doi: 10.1371/journal.pntd.0003215 (PMC4183468; doi:10.1371/journal.pntd.0003215)
Supplement: Table S3 — The amino acid sequences encoded in the DNA fragments inserted in the selected phage clones. Alignment of the peptides did not reveal a common motif. (DOCX) [file pntd.0003215.s004.docx]

**Table S3.** **The amino acid sequences encoded in the DNA fragments inserted in the selected phage clones.**

| **Gene** | **Peptide inserted in phage** |
| --- | --- |
| LIC10508 | IYGSDSVARSRHGRF |
| LIC11574 | ISILASTAANFIIPSGSDAAQTL |
| LIC12341 | FLNEGKYASETNRVKSIDPSI |
| LIC13411 | NERYNIKSQKLRYLSFYNIPVAERRRKKKEEFYKRFIEKQNLDRNDPAVQNALQNF |
